# European Consensus Guidelines on the Management of Respiratory Distress Syndrome: 2022 Update

**DOI:** 10.1159/000528914

**Published:** 2023-02-15

**Authors:** David G. Sweet, Virgilio P. Carnielli, Gorm Greisen, Mikko Hallman, Katrin Klebermass-Schrehof, Eren Ozek, Arjan te Pas, Richard Plavka, Charles C. Roehr, Ola D. Saugstad, Umberto Simeoni, Christian P. Speer, Maximo Vento, Gerry H.A. Visser, Henry L. Halliday

**Affiliations:** ^a^Regional Neonatal Unit, Royal Maternity Hospital, Belfast, UK; ^b^Department of Neonatology, University Polytechnic Della Marche, University Hospital Ancona, Ancona, Italy; ^c^Department of Neonatology, Rigshospitalet and University of Copenhagen, Copenhagen, Denmark; ^d^Department of Children and Adolescents, Oulu University Hospital and Medical Research Center, University of Oulu, Oulu, Finland; ^e^Department of Pediatrics and Adolescent Medicine, Division of Neonatology, Medical University of Vienna, Vienna, Austria; ^f^Department of Pediatrics, Marmara University Medical Faculty, Istanbul, Turkey; ^g^Leiden University Medical Centre, Leiden, The Netherlands; ^h^Division of Neonatology, Department of Obstetrics and Gynecology, General Faculty Hospital and 1st Faculty of Medicine, Charles University, Prague, Czechia; ^i^Faculty of Health Sciences, University of Bristol, UK and National Perinatal Epidemiology Unit, Oxford Population Health, Medical Sciences Division, University of Oxford, Oxford, UK; ^j^Department of Pediatric Research, Oslo University Hospital Rikshospitalet, University of Oslo, Oslo, Norway; ^k^Ann and Robert H. Lurie Children's Hospital of Chicago, Northwestern University Feinberg School of Medicine, Chicago, Illinois, USA; ^l^University of Lausanne, Lausanne, Switzerland; ^m^Department of Pediatrics, University Children's Hospital, Wuerzburg, Germany; ^n^Department of Pediatrics and Neonatal Research Unit, Health Research Institute La Fe, University and Polytechnic Hospital La Fe, Valencia, Spain; ^o^Department of Obstetrics and Gynecology, University Medical Centre, Utrecht, The Netherlands; ^p^Department of Child Health, Queen's University Belfast and Royal Maternity Hospital, Belfast, UK

**Keywords:** Antenatal corticosteroids, Continuous positive airway pressure, Evidence-based practice, Mechanical ventilation, Non-invasive respiratory support, Nutrition, Oxygen supplementation, Patent ductus arteriosus, Preterm infant, Respiratory distress syndrome, Surfactant therapy, Thermoregulation

## Abstract

Respiratory distress syndrome (RDS) care pathways evolve slowly as new evidence emerges. We report the sixth version of “European Guidelines for the Management of RDS” by a panel of experienced European neonatologists and an expert perinatal obstetrician based on available literature up to end of 2022. Optimising outcome for babies with RDS includes prediction of risk of preterm delivery, appropriate maternal transfer to a perinatal centre, and appropriate and timely use of antenatal steroids. Evidence-based lung-protective management includes initiation of non-invasive respiratory support from birth, judicious use of oxygen, early surfactant administration, caffeine therapy, and avoidance of intubation and mechanical ventilation where possible. Methods of ongoing non-invasive respiratory support have been further refined and may help reduce chronic lung disease. As technology for delivering mechanical ventilation improves, the risk of causing lung injury should decrease, although minimising time spent on mechanical ventilation by targeted use of postnatal corticosteroids remains essential. The general care of infants with RDS is also reviewed, including emphasis on appropriate cardiovascular support and judicious use of antibiotics as being important determinants of best outcome. We would like to dedicate this guideline to the memory of Professor Henry Halliday who died on November 12<sup>,</sup> 2022. These updated guidelines contain evidence from recent Cochrane reviews and medical literature since 2019. Strength of evidence supporting recommendations has been evaluated using the GRADE system. There are changes to some of the previous recommendations as well as some changes to the strength of evidence supporting recommendations that have not changed. This guideline has been endorsed by the European Society for Paediatric Research (ESPR) and the Union of European Neonatal and Perinatal Societies (UENPS).

## Introduction

Survival of preterm infants continues to improve, with those at 22–23 weeks of gestation now having reasonable chances of making it home to their families [1]. There is a growing trend towards offering initial life support to infants born at lower perceived levels of viability, which in some countries is now defined as low as 22 weeks of gestation [2]. However, we caution that recommendations given in these guidelines are based on evidence from studies, which almost without exception, have been generated in times where the resuscitation of the most preterm infants was not often attempted, and until very recently, infants of the lowest gestations were not included in clinical trials. We therefore recommend caution when applying current recommendations to the most preterm infants with respiratory distress syndrome (RDS). Until more evidence is available, these guidelines apply mainly to management of RDS in infants with gestational ages of greater than 24 weeks. Managing RDS remains a key component of neonatal intensive care. European data from 2014 to 2016 show that around 50% of all babies born between 22^+0^ and 32^+6^ weeks receive surfactant; therefore, relevant skills for surfactant administration and mechanical ventilation (MV) remain important [3].

RDS is caused by pulmonary immaturity and surfactant deficiency, resulting in respiratory insufficiency from soon after birth. There is now less emphasis on radiographic diagnosis and grading of RDS, such as “ground glass with air bronchograms.” Definitions based on blood gas analyses are also redundant, as management has moved towards an approach of pre-emptive treatment with surfactant based on clinical assessment of work of breathing and inspired oxygen requirement to avoid worsening RDS. The aim of modern RDS management is to maximise survival while minimising complications such as air leaks and BPD. Many strategies for prevention and treatment of RDS and providing early respiratory support have been tested in clinical trials and are summarised in updated systematic reviews, all of which inform these guidelines. This current version updates previous versions [4, 5, 6, 7, 8] after critical examination of the most recent evidence available to end of 2022. We have again used a format of summarising management strategies followed by evidence-based recommendations according to the GRADE system to reflect the authors' judgement of the evidence supporting each of the statements (Table 1). [9]. Summary of recommendations is shown in Table 2.

### Prenatal Care

Lack of antenatal care increases risk of death or severe morbidity [10]. General measures to reduce preterm birth include prevention of teenage pregnancies, adequate pregnancy spacing, prevention of unnecessary caesarean sections, early screening for preeclampsia and treatment with low-dose aspirin in women at risk, and single embryo transfer when in vitro fertilisation is used [11].

In asymptomatic pregnant women at risk of spontaneous preterm birth, due either to previous preterm birth or where a shortened cervix has been identified, use of progesterone is associated with a reduced rate of preterm birth and lower perinatal mortality [12]. This holds for singleton pregnancies only, as there are insufficient data on twins. Results for neonatal outcomes, including risk of RDS, were favourable but less certain [13]. Large trials have questioned the efficacy of progesterone in the absence of a short cervix, and there is very little evidence of benefit beyond the neonatal period. Routine cervical length measurements are advised in populations at risk of preterm birth and those with an overall low risk and/or very low incidence of short cervix, provided there is adequate quality assurance in measuring cervical length [12]. Cervical cerclage may also reduce preterm birth in high-risk singleton pregnancies [14]. The challenge is to identify high-risk pregnancies early and aim for effective prevention of preterm birth. The same holds for omega-3 fatty acid supplementation, which may also reduce preterm delivery [15] but most likely only in populations with poor nutrition.

Interventions to improve outcomes and prevent RDS begin before birth. There is often warning of impending preterm delivery and a need to consider interventions to prolong gestation or reduce risk of adverse outcomes by “preparing” the foetus. Cervical length measurement, possibly in combination with a biomarker [16], may determine which women are at risk of delivery within 7 days, perhaps allowing more judicious use of antenatal treatments. Preterm foetuses with expected delivery before 30 weeks of gestation should, where possible, be transported in utero to tertiary centres where appropriate skills are available; best outcomes are achieved for babies born in centres with a high throughput of VLBW babies [17]. In prenatal pre-labour rupture of membranes, antibiotics can delay preterm delivery and reduce neonatal morbidity, although co-amoxiclav should be avoided because of its association with increased risk of necrotising enterocolitis (NEC) [18]. Magnesium sulphate, given to women with imminent preterm delivery before 32 weeks, reduces incidence of cerebral palsy at 2 years by about 30%, although longer term benefits are unclear [19]. A reduction in cerebral palsy may be obtained if magnesium sulphate is given as close to 4 hours before delivery, so advanced dilatation is not a contraindication to treatment [19]. Overdosing must be avoided given maternal side effects such as vasodilatation and neuromuscular blockage. Tocolytic drugs can be used in the short term to delay birth, allow safe transfer to a perinatal centre, and allow prenatal corticosteroids time to take effect, although tocolytics have no direct beneficial effect on the foetus [20]. There is little evidence that delivering preterm infants by CS rather than allowing vaginal delivery improves outcome.

A single course of prenatal corticosteroids given to mothers with anticipated preterm delivery before 34 weeks of gestation improves survival; reduces RDS, NEC, and IVH; and is not associated with any significant maternal or short-term foetal adverse effects [21]. These favourable results were shown in the 1970s but also in clinical trials performed after 1990, indicating that even with modern neonatal care, prenatal steroids are beneficial. Prenatal corticosteroid therapy is recommended in all pregnancies with threatened preterm birth before 34 weeks of gestation where active care of the newborn is anticipated. Although there are limited RCT data in babies <25 weeks of gestation, observational studies suggest that antenatal corticosteroids, together with other active management practices, reduce mortality even down to 22 weeks [22], and current guidelines have incorporated these data in their recommendations [23]. In pregnancies between 34 and 37 weeks of gestation, prenatal steroids will also reduce risk of short-term respiratory morbidity, but not mortality, and there is increased risk of neonatal hypoglycaemia [24]; benefits reduce with increasing gestation, whereas the incidence of hypoglycaemia increases [25]. In women in spontaneous preterm labour after 34 weeks, steroid treatment is controversial and not advisable [26], as exposure is associated with a significantly higher risk of adverse neurocognitive and psychological outcomes [27]. Optimal treatment to delivery interval is more than 24 hours and less than 7 days. Beyond 7–14 days, benefits are diminished. In a cohort study, the beneficial effect of the first dose of antenatal steroids on very preterm infants starts within a day, so advanced dilatation is not a contraindication [28]. There is still debate about whether steroids should be repeated 1 or 2 weeks after the first course for women with threatened preterm labour. A repeat course reduces the risk of needing respiratory support but does not affect mortality or other serious health outcomes and reduces birth weight and head circumference [29]. The WHO recommends that a single repeat course of steroids be considered if preterm birth does not occur within 7 days after the initial course and there is a high risk of preterm birth in the next 7 days. None of the clinical trials showed any improved outcomes when the repeat course had been given after 32 weeks; a single repeat course should therefore be restricted to gestational age <32 weeks [30]. Most studies showing benefit include mixed populations of singleton and multiple pregnancies, but there are few studies on effects of antenatal corticosteroids focussing exclusively on multiple gestations. Steroids are potent drugs with many unwanted effects. When given appropriately, they improve outcome. If not, then side effects, such as dose-dependent impaired foetal length and head circumference, impaired placental growth, brain apoptosis, and increased infection risk, may prevail. Long-term follow-up of children from trials conducted in the 1970s has been reassuring; however, steroids given after 34 weeks are associated with impaired outcome. Recent data from Finland raise concerns that any prenatal steroid has a negative effect on neurological, cognitive, and behavioural disorders, especially for infants who are born at term [31]. Although these data were obtained from within sib-pair comparisons and logistic regression, they may be biased by the occurrence of preterm contractions, which, in itself, may be a risk factor for neurodevelopmental deficits [32].

Steroids should only be given to women who really are going to deliver preterm. This continues to be a challenge since 40–50% of women receiving steroids deliver at term [27]. Unnecessary use of steroids might be reduced by accurate dating of duration of gestation, preterm birth risk assessment, restriction of repeated courses to a single course before 32 weeks, avoidance of steroids in women at risk of late preterm delivery, and by avoidance of unnecessary early elective CS. The current antenatal steroid dosage appears to be high, and the release of betamethasone acetate proceeds slowly for several days [33]. However, a recent RCT from France, in which a single betamethasone injection was compared to two injections 24 h apart (*normal care*), showed a 2.5 percent reduced need for surfactant in the full betamethasone arm but no between-group differences in rates of mortality, or other important outcomes [34]. Maternal BMI might be a confounder, and further studies are needed. The same holds for studies in early foetal growth restriction, in which steroids are widely used without any randomised studies indicating their effectiveness.

### Recommendations

1. Mothers at high risk of preterm birth <28–30 weeks of gestation should be transferred to perinatal centres with experience in management of RDS (B1).

2. In women with a singleton pregnancy and a short cervix in mid-pregnancy or previous preterm birth, vaginal progesterone treatment should be used to increase gestational age at delivery and reduce perinatal mortality and morbidity (A1).

3. In women with symptoms of preterm labour, cervical length and accurate biomarker measurements should be considered to prevent unnecessary use of tocolytic drugs and/or antenatal steroids (B2).

4. Clinicians should offer a single course of prenatal corticosteroids to all women at high risk of preterm delivery, from when pregnancy is considered potentially viable up to 34 completed weeks of gestation, ideally at least 24 h before birth (A1).

5. A single repeat course of steroids may be given in threatened preterm birth before 32 weeks of gestation if the first course was administered at least 1–2 weeks earlier (A2).

6. MgSO_4_ should be administered to women with imminent delivery before 32 weeks of gestation (A1).

7. Clinicians should consider short-term use of tocolytic drugs in very preterm pregnancies to allow completion of a course of prenatal corticosteroids and/or in utero transfer to a perinatal centre (B1).

### Delivery Room Stabilisation

Birth is defined as when the foetus is completely expelled from the mother, and this is when timings should start [35]. Personnel attending birth should know how to identify infants who require urgent airway management and lung inflation in the first minutes after birth in order to establish gas exchange and restore cardiac output. European Resuscitation Guidelines [36] cover protocols for when and how to intervene in infants who are born in poor condition secondary to hypoxia, with emphasis on airway opening and aeration of the lungs using positive pressure. A separate approach is needed for most preterm babies at risk of RDS as they are not asphyxiated and with time will try to breathe on their own [37], and supporting normal transition with more gentle interventions is preferable.

The timing of umbilical cord clamping is an important first step. Deferring cord clamping (DCC), allowing time for a placental transfusion, and establishing lung aeration before clamping will lead to a better haemodynamic transition. Avoiding immediate cord clamping in preterm infants has been recommended since 2010, and recently, it is clear that DCC of 30–60 s will reduce both in-hospital mortality [38] and the combined outcome of death or major disability at 2 years [39]. Umbilical cord milking was recommended as an alternative where DCC is not possible. Recent systematic reviews suggest equivalence in terms of haemodynamic benefits and overall outcomes [40]; however, one randomised trial was stopped early because of an excess of severe intraventricular haemorrhage in the subgroup of infants below 28 weeks of gestation [41]. Stabilising preterm babies on specially adapted resuscitation tables that allow optimal thermal care and airway management with the umbilical cord intact is also feasible [42], resulting in more rapid establishment of airway support and longer duration of DCC without needing to move to a separate overhead warmer. However, it is still not clear if this confers any overall benefits to most infants who will begin spontaneous breathing before the cord is clamped.

Repetitive stimulation such as stroking the back may be helpful in terms of establishing regular respirations with improved saturations observed in the first minutes after birth [43]. Early caffeine therapy also might improve respiratory effort [44], though further studies are needed before firm recommendations can be made [45].

For spontaneously breathing preterm infants, CPAP initiation rather than intubation will reduce lung injury and BPD [46]. Traditionally, this has been managed using a face mask and a CPAP level of around 5–6 cm H_2_O; however, there is no strong evidence to recommend any particular level of pressure [47]. Routine sustained inflation of the lungs at birth should also not be performed as there is no evidence of benefit but evidence of harm in those <28 weeks [48]. T-piece devices allowing careful control of peak inspiratory pressures and PEEP are considered better than self-inflating bags. The interface between the CPAP device and the baby may also be important. Face mask application on its own may be sufficient to induce apnoea via the trigemino-cardiac reflex in spontaneously breathing infants [49]. Nasal interfaces offer an alternative method for CPAP provision at birth, but to date, no strong evidence exists that simply switching interface offers any clinical advantages [50]. Recently, a newly designed variable flow nasal prong CPAP delivery system reduced intubation requirements compared to standard T-Piece resuscitation with mask [51]. There is an urgent need for further work on improved interface design and strategies to provide early respiratory support to very preterm infants.

Heating and humidification of gases used for stabilisation is ideal in terms of preventing heat loss [52], but servo-controlled temperature regulation immediately after birth does not appear to confer any advantage [53]. Using plastic wraps and caps under radiant warmers for preterm babies <32 weeks will reduce hypothermia and result in less risk of IVH [52]; however, using these in combination with exothermic mattresses has the potential to cause overheating [54].

Significantly improved outcomes are found in newborn infants <32 weeks of gestation if SpO_2_ reaches 80–85% within 5 min [55]. Further, bradycardia and especially prolonged bradycardia (HR <100 bpm more than 2 min) combined with insufficient SpO_2_ (<80%) within 5 min increases the risk of death and IVH [56]. Monitoring infants' well-being during transition consists of assessment of adequate heart rate and saturations that are improving in line with normal values, increasing from 60% to 90% over the first 10 min after birth. It may take up to a minute to have reliable pulse oximetry readings for HR and SpO_2_. Heart rate can be rapidly counted by auscultation, which is not as accurate as oximetry or ECG [57]; however, it is not clear whether rapid ECG-derived HR signal confers any meaningful clinical benefit [58]. Caregivers are quite capable of determining when HR <60 or above 100 bpm which is all that is needed [59]. Respiratory function monitoring with flow sensors can also provide information during stabilisation that can identify leaks, airway obstruction, and tidal volumes graphically. A recent meta-analysis showed a decrease in IVH when respiratory function monitoring was used, but more research is needed before it can be routinely recommended [60].

Blended air/oxygen should be available. For term babies, resuscitation starting in air is best, but for preterm babies <32 weeks, it is still unclear what the ideal starting oxygen level should be, with no difference in major outcomes in randomised trials [61]. Prolonged bradycardia and hypoxia can occur in the smallest infants started in air, so the consensus is that starting with FiO_2_ around 0.30 for babies less than 29 weeks of gestation will give the best balance between avoiding hypoxaemia and causing oxidative stress [62]. A recent study has shown that starting with FiO_2_ 1.0 with careful titration was more successful in reducing risk of hypoxaemia, without increasing risk of hyperoxaemia, when compared to starting with FiO_2_ 0.30, and reducing hypoxaemia increased breathing effort [63]. Once transition has occurred and the infant is stable on CPAP, some centres would offer very early rescue surfactant to the smallest infants using the LISA technique in order to maximise the chance of avoiding MV [64].

When infants remain apnoeic and bradycardic, they need intubation for stabilisation, but this is in a minority of cases. Unfortunately, with increased use of non-invasive support, intubation skills are diminishing [65]. Regular simulation training with mannequins [66] and the use of video laryngoscopy for supervised intubations may help [67]. Delivery room intubations are usually indicated in infants born between 22 and 24 weeks and these or emergency intubations are usually performed without sedation [68]. If intubation is required, the correct placement of the endotracheal tube can be quickly verified by auscultation and by using a colourimetric CO_2_ detection device. Low tidal volume or HFOV and early surfactant administration prior to radiographic confirmation of RDS is now the recommended practice, based on observational studies [69].

### Recommendations

1. If clinical condition allows, defer clamping the umbilical cord for at least 60 s (A1). Only when DCC is not feasible, consider umbilical cord milking in infants with GA >28 weeks (B2)

2. T-piece devices should be used rather than bag and mask (B1)

3. Spontaneously breathing preterm infants should be stabilised using CPAP (A1). If apnoeic or bradycardic, start giving ventilation breaths. Expert consensus is to start with CPAP pressure at least 6 cm H_2_O and peak inspiratory pressures 20–25 cm H_2_O (D2)

4. Oxygen for resuscitation should be controlled using a blender. Use an initial FiO_2_ of 0.30 for babies <28 weeks of gestation and 0.21–0.30 for those 28–31 weeks, 0.21 for 32 weeks of gestation and above. FiO_2_ adjustments up or down should be guided by pulse oximetry (B2). SpO_2_ of 80% or more (and heart rate >100/min) should be achieved within 5 min (C2).

5. Intubation should be reserved for babies not responding to positive pressure ventilation via face mask or nasal prongs (A1).

6. Plastic bags or occlusive wrapping under radiant warmers and humidified gas should be used during stabilisation for babies <32 weeks of gestation to reduce the risk of hypothermia. Hyperthermia should also be avoided (A1).

### Surfactant Therapy

Surfactant therapy improves survival and reduces pneumothorax and therefore plays an essential role in management of RDS. Prior to 2013, *prophylactic* surfactant was recommended for the smallest babies as it improved survival in clinical trials from the pre-early CPAP era. Intratracheal surfactant administration requires skill and has the potential to cause harm, particularly when ventilating without controlling tidal volumes. Early initiation of CPAP may avoid the harmful effects of intubation and MV during the transitional phase, and since 2013, recommendations have been to only use surfactant in infants showing clinical signs of RDS. The overall aim is to avoid MV if possible while endeavouring to give surfactant as early as possible in the course of RDS, preferably using LISA methods.

#### Surfactant Administration Methods

Surfactant must be delivered directly to the trachea, and in most of the early trials, it was given as a bolus through an endotracheal tube, distributed by IPPV followed by a period of weaning ventilation. The IN-SUR-E technique, involving surfactant bolus administration followed by brief bag ventilation and rapid extubation without ongoing ventilation, seemed to reduce lung injury [70]. The accepted best method is to use a thin catheter for surfactant administration and avoid “bagging” completely, allowing the infant to maintain spontaneous breathing on CPAP while surfactant is gradually instilled in small aliquots. This method, known as less invasive surfactant administration (LISA) or minimally invasive surfactant administration, results in less need for MV and a reduction in the combined outcome of death or BPD as well as reduction in IVH in head-to-head comparisons with IN-SUR-E at identical treatment thresholds [71]. However, meta-analyses of multiple, individually small, non-blinded, underpowered studies may lead to over-estimating the benefits of LISA [72]. The largest study (OPTIMIST) randomised 485 babies of 25–28 weeks with blinding of the intervention: LISA surfactant versus sham procedure at FiO_2_ threshold 30%. Although there was no significant difference in the primary outcome of death or BPD, there was a significant reduction in BPD in survivors favouring the treated infants (37% vs. 45%) [73]. However, it is unclear if any differences can be attributed solely to the LISA method, as earlier compared to later surfactant in RDS is already known to be beneficial, and two-thirds of control infants received surfactant [73]. The LISA technique has been widely adapted in many parts of Europe and in large cohort studies there are better clinical outcomes [74]. LISA prophylaxis is also used for the smallest infants in some centres, although the benefits are still being assessed in clinical trials, and there are concerns about negative effects related to laryngoscopy including destabilisation that inevitably occurs during surfactant administration. A high level of competence for the LISA procedure, rescue intubation, and CPAP application is required [75]. Two-year follow-up from one of the larger randomised trials also gives reassurance that the LISA technique is safe [76]. Early rescue with LISA also has potential to reduce overall costs of care [77].

Laryngoscopy for LISA surfactant is undoubtedly uncomfortable, but there is more chance of apnoeic episodes post-procedure requiring PPV if sedation is used [78]. In practice, the ease of the procedure seems unaffected whether opiates, oral sucrose, or no sedation is used [79]. Further clinical trials are underway to assess benefits and risks of sedation for LISA surfactant (NCT05065424). Alternative methods of getting adequate surfactant doses into the lung in a gentler way would be ideal. Laryngeal masks can be used to administer surfactant in babies [80]. A recent study confirmed non-inferiority to IN-SUR-E in preterm babies as low as 800 g, probably related to sedation protocols for endotracheal tube placement [81].

Modern nebulisers are capable of aerosolising surfactant, but to date, studies on nebulisation have not convincingly shown any meaningful improvement in smaller infants who should benefit most [82]. Pharyngeal deposition of surfactant at birth does not result in any clinical improvements [83].

#### When to Treat with Surfactant?

If intubation is deemed necessary as part of stabilisation for preterm infants, then surfactant should be given to promote early extubation [84]. Most preterm infants will transition successfully on CPAP, but those with RDS will develop progressively worsening lung disease, clinically presenting as increased work of breathing, sternal recession, and increasing oxygen requirements to maintain normal saturations. Following the natural course of RDS, spontaneous recovery usually begins after 48–72 h, and infants with milder disease may manage without surfactant, thereby avoiding the discomfort of laryngoscopy and potential deleterious effects of intubation. Early trials showed that surfactant given earlier in the course of disease works better than later in terms of reducing air leaks in babies ventilated for RDS [84] and avoiding MV if the IN-SUR-E technique is used [85]. This creates a dilemma for neonatologists as at present RDS severity is determined clinically, using a combination of FiO_2_ requirements, coupled with judgement of work of breathing and other signs of respiratory distress alongside the degree of lung aeration on chest radiograph (or ultrasound), all of which can be influenced by CPAP. Ideally, predicting surfactant deficiency before the infant has deteriorated would enable earlier surfactant therapy of infants on CPAP and is likely to result in less need for MV and improved outcomes. Our previous recommendation to use FiO_2_ > 0.30 as the threshold for surfactant treatment was based on observations of CPAP failure rates according to early postnatal oxygen requirements and is supported by more recent data [86]. In these studies, FiO_2_ at 2 h of life was used to predict later CPAP failure, which was defined as oxygen requirement of 50–60%. In a newer similar study, CPAP failure was defined as oxygen requirement of 30% and thus optimal prediction was obtained at a 2-h FiO_2_ as low as 23% [87]. As RDS is typically progressive over the first days of life, it is no surprise that FiO_2_ cut-off for surfactant administration should be age specific. Furthermore, the use of early nasal ventilation and the knowledge that simply increasing mean airway pressure is likely to lower FiO_2_ requirements, even in surfactant-deficient infants, contribute to the debate of optimal FiO_2_ cut-off [88]. Lung ultrasound, with appropriate training, may offer an alternative way of diagnosing RDS at an earlier stage, without apparently resulting in more infants overall being treated [89]. Rapid bedside testing for surfactant components in gastric aspirate is also now available, and clinical trials of this new point-of-care test to determine surfactant need at birth are underway [90].

The dilemma as to whether to treat late-preterm and early-term infants with RDS with surfactant is being addressed in the SURFON Trial (https://www.npeu.ox.ac.uk/surfon). The current evidence for more mature infants with signs of RDS indicates a potentially decreased risk of mortality, air leaks, persistent pulmonary hypertension, and duration of respiratory support. However, due to heterogeniety of data, there is currently not enough evidence to make any recommendations [91].

#### Repeated Surfactant Dosing

More than one dose of surfactant may be needed. Many infants can continue on NIV even when surfactant is required. If poractant alfa is used, need for re-dosing can be minimised by using a larger initial dose of 200 mg/kg [92]. For other surfactants, such data are not available.

#### Surfactant Preparations

Three natural (animal derived) surfactants are currently available in Europe. Beractant (Survanta) at recommended dose of 100 mg/kg requires surfactant dose volume of 4 mL/kg. Bovactant (Alveofact) at recommended dose of 50 mg/kg requires volume of 1.2 mL/kg. Poractant alfa (Curosurf) at recommended dose of 100–200 mg/kg requires dose volume of 1.25–2.5 mL/kg. Head-to-head trials show similar efficacy among surfactants when used in similar doses; however, there is a survival advantage when poractant alfa at the higher dose of 200 mg/kg is compared to 100 mg/kg of poractant alfa or beractant [93]. New synthetic surfactants with surfactant protein analogues have shown promise in clinical trials, potentially negating the need for animals in surfactant production, but these are not yet commercially available [94]. Using surfactant with added budesonide may also reduce BPD [95]. Further studies are underway, and we should await the outcomes of these before recommendations are made (https://www.plusstrial.org).

### Recommendations

1. If a preterm baby <30 weeks of gestation requires intubation for stabilisation, they should be given surfactant (A2).

2. Babies with RDS needing treatment should be given an animal-derived surfactant preparation (A1).

3. LISA is the preferred method of surfactant administration for spontaneously breathing babies on CPAP (A1).

4. Laryngeal mask surfactant may be used for more mature infants >1.0 kg (B2).

5. An initial dose of 200 mg/kg of poractant alfa is better than 100 mg/kg of poractant alfa or 100 mg/kg beractant for rescue therapy (A1).

6. Rescue surfactant should be given early in the course of the disease (A1). Suggested protocol would be to treat worsening babies with RDS when FiO_2_ > 0.30 on CPAP pressure ≥6 cm H_2_O or if lung ultrasound suggests surfactant need (B2).

7. A second and occasionally a third dose of surfactant should be given if there is ongoing evidence of RDS such as persistent high oxygen requirement and other problems have been excluded (A1).

### Oxygen Supplementation beyond Stabilisation

There are no relevant changes since 2019 in terms of refining previous recommendations for oxygen saturation targeting based on data from the NeOProm Collaboration [96]. Targeting lower saturations (85–89% vs. 91–95%) reduces risk of severe retinopathy of prematurity (ROP) but at expense of increasing mortality (RR 1.17; 95% CI: 1.04–1.31) and NEC. Recommendations therefore remain the same, targeting saturations between 90 and 94% by setting alarm limits between 89 and 95%, although ideal oxygen saturation targets are still unknown, particularly for preterm babies above 30 weeks [97]. There are no scientific data supporting the choice of alarm limits. We argue for tight alarm limits to prevent fluctuations in oxygenation and avoid hypoxaemia and or hyperoxaemia. Recent publications have emphasised an increased incidence of ROP associated with higher oxygen saturation ranges in the NICU [98]. Therefore, it is recommended that there be stringent surveillance for prevention and early treatment of ROP, especially in preterm infants at the lowest gestational ages. Recently, a Swedish study confirmed that lowering saturation targets is associated with less ROP but greater risk of NEC and higher mortality [99].

Approximately 20% of infants with BPD will develop pulmonary hypertension. The optimal oxygen saturation targeting strategy to prevent and/or support extremely preterm infants with pulmonary hypertension remains unclear. However, a 50% reduction in BPD-associated pulmonary hypertension was observed in babies <29 weeks of gestation after oxygen saturation targets were increased from 88–92% to 90–95%, supporting use of higher saturation targets in these infants [100].

Reliability of servo-controlled algorithms for keeping oxygen saturations within defined ranges has been tested in the delivery room [101] as well as in MV [102] and NIV neonates [103]. Automated oxygen control significantly increases the time within the intended range, thus potentially reducing nursing workload. However, long-term clinical benefits are yet to be determined.

### Recommendations

1. In preterm babies receiving oxygen, the saturation target should be between 90 and 94% (B2).

2. Alarm limits should be set to 89% and 95% (D2)

3. Protocols for screening and treating preterm babies for ROP should be in place (A1)

### Non-Invasive Respiratory Support

Preterm infants should be managed without MV where possible and if needed, time on MV should be minimised. CPAP has been successfully used for >50 years for stabilising preterm infants, in both high- and middle-to-low-income settings [104]. CPAP improves lung volume, especially functional residual capacity. The increased positive distending airway pressure improves oxygenation, decreases apnoea, and reduces the work of breathing. Thus, CPAP is recommended as the first choice for primary and secondary respiratory support [105, 106, 107]. However, alternative modes of NIV are increasingly being tested in clinical trials against CPAP as the gold standard.

CPAP involves delivering gas, ideally heated and humidified, with a measurable and controllable pressure which is transmitted using an interface such as short soft nasal prongs or mask, connected tightly to the baby's face creating a seal. CPAP pressures are typically set between 5 and 9 cm H_2_O. Increasing airway pressure provides several benefits including splinting the upper airway, maintaining lung expansion, and preventing end-expiratory alveolar collapse. Other benefits include reduced apnoea rates, improved tidal volumes, higher functional residual capacity, and reduced work of breathing. Higher pressures improve oxygenation but potentially increase risk of air leaks. An underwater seal or “bubble CPAP,” to generate the pressure, causes small fluctuations around the set pressure which some believe offers the additional advantage of improved CO_2_ washout [108]. When weaning babies from CPAP, a gradual reduction in pressure rather than sudden cessation of CPAP results in greater likelihood of success [109].

Using a flow driver to generate CPAP has the theoretical advantage of offloading expiratory work of breathing, although no important clinical differences have been shown in clinical trials among various CPAP devices. The simplicity of bubble CPAP systems allows their use in low-income settings with some evidence of benefit over free-flowing oxygen [104]. Leaks between the interface and nose are common with both nasal prongs and masks but somewhat less with prongs [110]. All CPAP interfaces carry risk of facial distortion and nasal trauma [111].

Bi-level CPAP, Duo-PAP, or BIPAP are variants between CPAP and IPPV that use low pressure differences between inspiratory and expiratory phases at PIPs of 9–11 cm H_2_O at rates of around 20–40 per minute with long inspiratory times of up to 1.0 s. There is no evidence that BIPAP confers any advantage over CPAP, and any clinical differences may simply reflect a higher overall mean airway pressure [112]. Modern ventilators provide NIPPV using pressures similar to those used for invasive MV. Challenges of NIPPV relate to pressure delivery through a non-sealed system, which is limited by leak at the nasal interface and the infant's tolerance to gas inflation of the stomach. Ventilator inflations can be synchronised with the infant's breathing by using either an abdominal capsule or in-line sensors. Synchronisation of nasal ventilation further improves respiratory stability [113]. Recent systematic reviews comparing different modes of NIV for primary respiratory support or post-extubation concluded that synchronised NIPPV was the most effective, decreasing the need for MV, or re-ventilation, in preterm infants [114, 115].

Supraglottic application of nasal HFOV (nHFOV) is used in some centres in Europe [116] and is the subject of ongoing research [117]. The oscillations are apparently transmitted all the way down to the lungs [118]. Regional ventilation may be similar between nHFOV and CPAP, while end-expiratory lung volume may be higher and aeration homogeneity slightly improved during nHFOV [119]. To date, four RCTs have compared nHFOV to CPAP, and a systematic review suggests that nHFOV decreases intubation rates when compared to CPAP [118]. However, there is lack of clarity in the methodology of some of the studies included as to how nHFOV was performed, making it difficult for studies to be replicated and for firm recommendations to be made.

Heated humidified high flow nasal cannulae (HFNC) are being increasingly used as an alternative to CPAP [120]. With HFNC, heated/humidified gas is delivered to the nostrils with nasal catheters that are specifically designed not to occlude the nostrils. Typically, flows of between 2 and 8 L/min are used, with weaning of flow rate determined clinically by FiO_2_ remaining low and a judgement of work of breathing [121]. Although HFNC undoubtedly generates some pharyngeal distending pressure, this is not measured, and the primary mode of action is likely related to gas conditioning and nasopharyngeal dead space CO_2_ washout [121]. In clinical trials, HFNC is broadly observed as equivalent to CPAP for babies >28 weeks coming off MV, with greater ease of use and less nasal trauma [122], although there is less evidence for smaller babies. In a large-scale non-inferiority RCT, HFNC more often resulted in treatment failure compared to CPAP, but the need for MV was similar [123], and inferiority related to this predefined primary outcome should not necessarily be equated with futility [124]. Clinicians from centres familiar with both HFNC and CPAP argue that with growing experience, HFNC can be used as initial support strategy, even in the smallest babies, and this is supported by recent systematic reviews [125, 126]. Acknowledging the many positive attributes of HFNC, including less nasal trauma, reduced pneumothorax, greater patient satisfaction, and parent and nursing staff preference, a more recent meta-analysis of HFNC trials recommends HFNC use as a first-line option for respiratory support in centres capable of offering CPAP and/or NIPPV as a backup [126]. There will likely be further refinements of NIV over the coming years. Better synchronisation with the baby's own breathing efforts may be a central element of modern ventilatory support. This could potentially be achieved by using the neurally adjusted ventilator assistance technology [127]. A recent small RCT suggests NIV-NAVA may be effective in preventing extubation failure in preterm infants, with lower PIP following extubation; however, the study was not powered to look at important clinical outcomes [128]. Larger clinical trials of NAVA and other newer modes of support are urgently needed.

### Recommendations

1. CPAP or (s)NIPPV should be started from birth in all babies at risk of RDS, such as those <30 weeks of gestation who do not need intubation for stabilisation (A1).

2. NIV with early rescue surfactant by LISA technique is considered optimal management for babies with RDS (A1).

3. The system delivering CPAP is of little importance; however, the interface should be short binasal prongs or mask with a starting pressure of about 6–8 cm H_2_O (A2). Ability to escalate to NIPPV will reduce the need for invasive MV in some infants (A1).

4. BIPAP devices confer no advantage over CPAP alone (A2). However, synchronised NIPPV, if delivered through a ventilator, can reduce need for ventilation or need for re-ventilation following extubation and may reduce BPD (A2).

5. HFNC can be used as an alternative to CPAP for some babies, with the advantage of less nasal trauma, provided centres have access to CPAP or NIPPV for those failing this mode (B2).

### MV Strategies

Despite best efforts to maintain as many preterm babies as possible on NIV, around half of babies <28 weeks will require MV and those that do have worse outcomes [129]. In addition, around half of infants <28 weeks will also fail their first attempt at extubation and they too have worse outcomes [130]. It is important that those managing infants with RDS understand the principles of MV to minimise the risk of iatrogenic lung injury. The aim of MV is to provide “acceptable” blood gases by ventilating at optimal lung volumes (*open lung concept*) while avoiding over-distension and atelectasis. Pulmonary over-distension increases risk of air leaks and pulmonary interstitial emphysema, while ventilating at sub-optimally low pressures may cause atelectasis and repeated pulmonary sheer stress, which in turn can generate inflammation and lung injury.

Modern ventilators have flow sensors that measure the volume of gas entering and leaving the endotracheal tube to help synchronise MV with the infant's own breathing efforts and may be used to limit the support provided to prevent lung over-distension. Volume-targeted ventilation (VTV) allows real-time weaning of pressure as lung compliance improves and results in less time on ventilation, fewer air leaks, and less BPD [131]. Setting an initial tidal volume of around 5 mL/kg with a maximum PIP at a safe level, around 25–30 cm H_2_O, with adjustments in initial tidal volumes according to judgement of work of breathing and blood gas assessment is usually straightforward but may fail if the endotracheal tube leak is high. Adjusting PEEP to maintain an open lung is judged by finding a point where FiO_2_ is at its lowest with haemodynamic stability and acceptable blood gases. Required tidal volumes vary around 5–7 mL/kg; the range tends to increase with increasing postnatal age [132]. Ventilation modes supporting every spontaneous breath rather than synchronised IMV makes sense, although if volume targeting is not possible, it may be safer to use synchronised IMV where the ventilation rate is clinician controlled [133]. Volume targeting can reduce hypocarbia in even the smallest infants [134]. Supporting the infants' own respiratory efforts with modes where both inspiration and expiration are synchronised, such as pressure support or NAVA, can improve patient comfort and facilitate weaning. However, to date, no differences have been shown in longer term clinical outcomes [135, 136].

HFOV is a ventilation strategy that facilitates gas exchange by very small gas volume delivery at fast rates to an optimally inflated lung, using a continuous distending pressure (CDP). Meta-analysis of studies comparing HFOV to conventional ventilation shows a modest reduction in BPD; however, there is a paucity of data from contemporary trials, comparing HFOV to VTV [137]. Determining the optimal distending pressure clinically involves finding the pressure at which oxygenation deteriorates during a stepwise reduction from full lung inflation and aiming for 1–2 cm H_2_O above this, bearing in mind that as compliance improves, the lung may become over-distended if the set pressure is not weaned [138]. Volume targeting in HFOV reduces CO_2_ variability [139] and allows effective ventilation with very small tidal volumes, which may be lung protective. Whatever ventilation system is used within an individual unit, it is important that all staff should be familiar with it.

Inhaled nitric oxide (INO) is a proven therapy for hypoxaemic respiratory failure in term babies with pulmonary hypertension. Although there is no significant effect of INO on mortality or BPD in preterm infants <34 weeks of gestational age [140], it is still often used on a physiological rationale during hypoxic respiratory failure. INO should not be used as a panacea for infants with poor oxygenation since INO is a toxic oxidant. However, for a small subgroup of preterm infants, with a history of mid-trimester oligohydramnios, birth asphyxia, and documented pulmonary hypertension with severe respiratory distress, brief NO treatment can result in rapid improvement in oxygenation, allowing ventilator settings to be reduced to safer levels [141].

At initiation of MV, lung recruitment may be used to optimise PEEP, but there is little evidence it influences outcome [142]; however, lung recruitment before surfactant administration may lead to successful early extubation [143]. Once stabilised on MV and with demonstrable spontaneous breathing effort, clinicians should immediately start planning for weaning to NIV [144]. Some infants require a very short period of ventilation, particularly those with RDS following surfactant therapy, and early extubation of even the smallest babies who achieve low ventilator settings should be encouraged. Infant's size, absence of growth restriction, oxygen requirement, and blood gases can all help determine extubation success [130]. Delaying extubation does not improve the chance of success [145]. Trials of endotracheal tube CPAP to predict extubation readiness are not that helpful [146]. Mathematical models for predicting extubation success may be useful, but their potential to improve outcomes has yet to be evaluated [147]. Extubation is possible from when MAP reaches about 7–8 cm H_2_O on conventional ventilation or a CDP of 8–9 cm H_2_O on HFOV. Extubating to a relatively higher CPAP pressure of 7–9 cm H_2_O or NIPPV will improve chance of success [148], although at present, there are no data to support any particular CPAP level in terms of influencing longer term outcomes [47].

#### Caffeine Therapy

Methylxanthines are respiratory stimulants, and caffeine therapy is now a well-established aspect of newborn respiratory care. The Caffeine for Apnea of Prematurity (CAP) study confirmed that caffeine in the standard dose of 20 mg/kg loading with 5–10 mg/kg day maintenance for infants <1,251 g coming off MV, or being treated for apnoea, resulted in less need for respiratory support, subsequently less BPD, and long-term follow-up also showing improved neurodevelopmental outcomes [149]. Subgroup analysis from the CAP trial showed better outcomes with earlier treatment [150]. Caffeine prophylaxis has become widespread based on cohort studies, *despite the risk of bias*, since earlier treatment is associated with better outcomes [150, 151]. Randomised trials of caffeine prophylaxis for extremely preterm infants are scarce and show conflicting results [152, 153], and further studies combining caffeine prophylaxis with LISA are currently underway. Higher doses may further improve respiratory outcomes but are associated with risk of adverse effects such as seizures or cerebellar haemorrhage. Gradual increase in dosing from 5 towards 8 mg/kg/day over several weeks may give the best chance of maintaining therapeutic effect [154].

#### Permissive Hypercapnia

The concept of facilitating earlier extubation by tolerating mild hypercapnia is long-standing [155]. Recent systematic review suggests the safest range for CO_2_ is around 5–7 kPa, with hypocapnia in preterm infants being associated with increased risk of periventricular leukomalacia and severe hypercapnia linked with IVH, NEC, BPD, and ROP [156]. Permissive hypercapnia will potentially allow reduced tidal volumes and facilitate extubation, though there is no convincing evidence that it reduces BPD. The optimal CO_2_ target remains to be determined; however, the consensus view is that tolerating modest degrees of hypercarbia is reasonable, provided the pH is acceptable.

#### Postnatal Steroids

Despite best efforts, some infants are difficult to wean from MV with an apparent cycle of MV-induced lung inflammation and increased risk of BPD. Systemic corticosteroids have a role in breaking this cycle to facilitate extubation and improve outcomes [157, 158]. However, steroids are powerful drugs, increasing risk of gastrointestinal perforations, and have potential to increase risk of long-term neurological problems, particularly if used within the first week of life. Meta-regression analysis suggests that the greater the risk of BPD, the more the balance tips in favour of using steroids to improve overall long-term outcomes [159]. The lower dose dexamethasone regimen described in the DART trial seems to get the best balance of clinical effectiveness with minimising risk of longer term side effects [160, 161]. Low-dose prophylactic hydrocortisone for 10–15 days from birth also improves chances of survival without BPD and reduces need for PDA treatment [162]. Concerns about effects on brain growth in observational cohorts exposed to postnatal hydrocortisone can be explained by the infants being sicker, and ventilated for longer, rather than a direct effect of hydrocortisone per se [163]. Some centres have already adopted routine early hydrocortisone supplementation to improve perinatal outcomes. However, early routine hydrocortisone may have negative interaction with potential high levels of endogenous cortisol (if not measured), which may increase the risk of severe IVH and spontaneous intestinal perforation [164]. Hydrocortisone prophylaxis seems promising, but more work is required before it is routinely adopted for all preterm infants, especially those below 26 weeks of gestation [165]. Prophylactic inhaled corticosteroids would be a logical alternative, and there is evidence of a reduction in BPD if inhaled budesonide or fluticasone is started from birth [166, 167]. The largest study of inhaled budesonide showed inexplicable higher mortality in the inhaled steroid-treated group, thereby prompting caution in recommending routine inhaled steroid prophylaxis [168, 169].

#### Pain and Sedation

Preterm babies can clearly experience pain and discomfort. There is a balance between appropriate analgesia and the negative effects of sedation, particularly when there is an emphasis on minimising duration of MV. Routine sedation for ventilated infants is not recommended [170]. Delivery room endotracheal intubations are often emergencies and not usually performed under sedation [68]. However, for elective intubations in the NICU, sedation with an opioid and muscle relaxant results in greater intubation success on the first attempt [171]. Sedation for LISA is discussed in the surfactant section.

### Recommendations

1. MV should be used in babies with RDS when other methods of respiratory support have failed (A1). Duration of MV should be minimised (B2).

2. Lung-protective modes such as VTV or high-frequency oscillation ventilation should be the first choice for babies with RDS who require MV (A1).

3. When weaning from MV, it is reasonable to tolerate a modest degree of hypercarbia, provided the pH remains above 7.22 (B2). Avoid pCO_2_ < 4.7 kPa (35 mm Hg) when on MV to reduce brain injury (C1).

5. INO in preterm babies should be limited to a therapeutic trial for those in whom there is documented pulmonary hypertension with severe respiratory distress and stopped if there is no response (D2).

4. Caffeine (20 mg/kg loading, 5–10 mg/kg maintenance) should be used to facilitate weaning from MV (A1). Early caffeine can be considered for babies at high risk of needing MV such as preterm babies on NIV (C1).

5. A short tapering course of low-dose dexamethasone should be considered to facilitate extubation in babies who remain on MV after 1–2 weeks (A2).

6. Opioids should be used selectively when indicated by clinical judgement and evaluation of pain indicators (D1). The routine use of morphine or midazolam infusions in ventilated preterm infants is not recommended (A1).

#### Monitoring and Supportive Care

Monitoring physiological variables in preterm babies is an important part of quality care. Pulse oximetry from birth can help titrate oxygen. In the NICU, there should be access to continuous pulse oximetry and ECG monitoring and a means of measuring PaCO_2_. Detection of exhaled CO_2_ by colourimetric devices can confirm correct placement of endotracheal tubes. Continuous end-tidal CO_2_ confirms ongoing correct tube placement as well as showing trends in gas exchange. Transcutaneous oxygen and CO_2_ monitoring can give continuous information for trending, but readings can be affected by other conditions such as sepsis [172]. Arterial blood gases are the gold standard, and umbilical or peripheral arterial cannulation is necessary if regular blood gases or if continuous blood pressure monitoring is required. Methods of monitoring cerebral oxygenation by near-infrared spectroscopy are available to enable clinicians to detect and reverse cerebral hypoxia [173], although whether this improves outcomes is still to be tested in large clinical trials. Close monitoring of haematological values and electrolytes ideally using very small volumes of blood is essential. There should be access to portable ultrasound and round-the-clock access to radiology services, which are needed to confirm RDS diagnosis and ensure correct placement of tubes and lines.

#### Temperature Control

Maintaining normal body temperature is an important quality measure as admission hypothermia is associated with worse outcomes, regardless of hospital performance [174]. In newborn preterm infants, immediate wrapping in a food-grade polythene bag or foil, placement under a radiant warmer, and humidification of gases are proven effective measures for reducing heat loss. After admission, babies should be managed in servo-controlled incubators, initially with relatively high humidity which can be reduced as skin integrity improves. Periods of skin-to-skin care are also an effective means of maintaining temperature and should be encouraged as they maximise bonding and improve growth and breastfeeding rates for VLBW infants [175].

#### Antibiotics

Mothers are often treated with intrapartum antibiotic prophylaxis if they present in preterm labour to reduce risk of group B streptococcal infection in the infant [176]. Antibiotics are also frequently started in babies with RDS until sepsis has been excluded, and it is important to have policies in place to narrow the spectrum and minimise duration, as antibiotic overuse is associated with adverse effects such as increased risk of NEC [177]. Guidelines should be in place to only screen for sepsis when there are additional risk factors or signs of sepsis, and it is reasonable to withhold antibiotics in low-risk scenarios such as infants born by elective caesarean section. If antibiotics are started empirically, it should be possible to stop them within 36 h if there is no clinical or laboratory evidence of sepsis [178].

#### Early Fluids and Nutritional Support

The smallest infants have very high initial transcutaneous water losses and water and sodium move from the interstitial to the intravascular compartments, making fluid balance challenging. Humidified incubators rather than overhead warmers help reduce insensible losses. Fluids are initiated at about 70–80 mL/kg/day and adjustments individualised according to fluid balance, weight change, and serum electrolyte levels. A modest early postnatal weight loss is normal. Regimens with more restricted fluids have better outcomes with reductions in PDA, NEC, and BPD [179]. Delaying introduction of sodium supplementation until beyond the third day or 5% weight loss will also improve outcome [180]. Parenteral nutrition should be started immediately as enteral feeding is initially limited. Early initiation of higher levels of parenteral amino acids results in less postnatal growth failure and an increase in positive protein balance [181]. At least 1.5 g/kg intravenous protein and 1–2 g/kg lipids should be started from day 1 and increased to a maximum of 3.5 g/kg amino acid [182, 183]. For stable infants, a small amount (0.5–1 mL/kg/h) of breast milk can be started early to initiate enteral feeding [184]. There is no evidence of increased NEC with advancing feeds fairly rapidly up to 30 mL/kg/day in stable VLBW babies [185]. Mother's milk is the preferred option for initiation of feeding but if not available then pasteurised donor breast milk is better than formula for reducing risk of NEC but will result in slower postnatal growth [186].

### Recommendations

1. Core temperature should be maintained between 36.5°C and 37.5°C at all times (C1).

2. Most babies should be started on intravenous fluids of 70–80 mL/kg/day in a humidified incubator, although some very immature babies may need more (C2). Fluids must be tailored individually according to serum sodium levels, urine output, and weight loss (D1).

3. Parenteral nutrition should be started from birth. Amino acids 1.5–2 g/kg/d should be started from day 1 and quickly built up to 2.5–3.5 g/kg/d (B2). Lipids 1–2 g/kg/d should be started from day 1 and quickly built up to 4.0 g/kg/day as tolerated (C2).

4. Enteral feeding with mother's milk should be started from the first day if the baby is hemodynamically stable (B2).

5. In infants with RDS, antibiotics should be used judiciously and stopped early when sepsis is ruled out (D1).

### Managing Blood Pressure and Perfusion

Hypotension and low systemic blood flow are associated with adverse outcomes, although thresholds for treating low blood pressure are unclear and very difficult to study [187]. Hypotension is associated with cerebral hypoxia, and the longer the hypoxia, the greater the risk of IVH; however, increasing blood pressure using dopamine may not change cerebral oxygenation [188]. Normal values are available showing lower blood pressure with decreasing gestation but increasing gradually over the first days and a wide variation at each gestational age [189]. Higher mean blood pressure after birth is achieved with antenatal steroid use, delayed cord clamping, and avoidance of MV. Neonatologist-performed functional echocardiography requires training but allows assessment of the cause of hypotension such as hypovolaemia, ductal shunting, or poor myocardial contractility [190]. Hypovolaemia is probably over-diagnosed, and administration of saline boluses is associated with poorer outcomes [191]. Dopamine is more effective than dobutamine at increasing blood pressure in hypotensive infants, although adrenaline may be a more rational choice in the setting of reduced ventricular function [192]. Hydrocortisone is also an effective inotrope and should be considered for extremely preterm infants who are at risk of low serum cortisol [193].

All infants start life with a patent (open) ductus arteriosus (PDA), and most will close spontaneously, but persistence of PDA and its potential adverse effects remain highest among the least mature infants. Hemodynamically significant PDA increases pulmonary oedema and decreases systemic cardiac output as the PVR gradually falls after birth. Although CDP reduces lung oedema and inotropic drugs can improve cardiac contractility, early closure of ductus remains desirable. PDA requiring medical closure is associated with increased risk of death, BPD, IVH, and NEC. Recent large cohort studies suggest that active early screening and treatment of PDA can reduce pulmonary haemorrhage and in-hospital mortality [194] and lower the risk of BPD [195]. Ibuprofen, indomethacin, or paracetamol induce closure of PDA, and differences in efficacy among the individual drugs or the administration route may be due to population variation [196, 197, 198]. Ibuprofen and particularly indomethacin increase risk of gastrointestinal perforation and bleeding and risk of renal insufficiency. Although paracetamol may cause hepatic and pulmonary toxicity due to oxidant injury, and antenatal exposure may predispose to neuro-psychiatric problems in early childhood, the few follow-up studies of infants receiving paracetamol soon after birth have failed to confirm significant adverse effects [199]. Routine treatment of preterm babies to promote PDA closure is not considered good practice [200]. Expectant management of PDA versus treatment is being explored in clinical trials [201] as well as early treatment with paracetamol. Surgical ligation has a place only if medical therapy has failed and the PDA is causing significant clinical problems [202].

Maintaining a reasonable Hb concentration is also important and late cord clamping contributes towards this goal. Randomised trials comparing targeting lower versus higher Hb concentrations (about 1–2 g/dL lower) show that targeting lower transfusion thresholds result in reduced need for blood transfusion without affecting hospital outcomes, and recent, major randomised controlled trials found similar long-term outcomes [203, 204]. The thresholds proposed in these guidelines therefore approximate the lower thresholds used in these trials [205].

### Recommendations

1. Treatment of hypotension is recommended when there is evidence of poor tissue perfusion such as oliguria, acidosis, and poor capillary refill (C2). Treatment will depend on the cause.

2. When a decision is made to attempt pharmacologic closure of hemodynamically significant PDA, indomethacin, ibuprofen, or paracetamol can be used with a similar efficacy (A2). Paracetamol is preferred when there is thrombocytopaenia or concerns about renal function (B2).

3. Thresholds for red blood cell transfusion in infants can be set at 12 g/dL (HCT 36%) for those with severe cardiorespiratory disease, 11 g/dL (HCT 30%) for those who are oxygen dependent, and 7 g/dL (HCT 25%) for stable infants beyond 2 weeks of age (A2).

### Miscellaneous

Therapy-resistant severe respiratory distress due to malformations, alveolar-capillary dysplasia, damaging mutations including SP-B, ABCA3, or TTF-1, or due to extreme structural immaturity is beyond the scope of these guidelines. On the other hand, surfactant therapy is reported to be useful in other serious situations where secondary surfactant inactivation occurs such as in pneumonia [206], pulmonary haemorrhage [207], or meconium aspiration syndrome [208]. Surfactant has also been trialled in preterm infants with evolving BPD who remain on MV at 2 weeks of age. Although there was no reduction in BPD at 36 weeks, surfactant-treated infants had fewer episodes of rehospitalisation over the first year of life [209]. Routine surfactant therapy is not recommended in infants with congenital diaphragmatic hernia [210].

### Recommendations

Surfactant can be used for RDS complicated by congenital pneumonia (C2).
Surfactant therapy can improve oxygenation following pulmonary haemorrhage (C1).
Surfactant can improve oxygenation in infants with severe meconium aspiration syndrome (B2).

## Conflict of Interest Statement

Henry L. Halliday, Christian P. Speer, and Charles C. Roehr in the past have been consultants to Chiesi Farmaceutici, Parma, the manufacturer of a leading animal-derived surfactant preparation used to treat RDS and a caffeine product for treatment of apnoea of prematurity. Virgilio Carnielli is a member of the Chiesi Farmaceutici Advisory Board. Henry Halliday and Christian Speer are joint chief editors of *Neonatology*.

## Funding Sources

This project was supported by an unrestricted educational grant provide by Chiesi Pharmaceuticals through UENPS to support travel for a single face-to-face meeting in Belfast during guideline preparation.

## Author Contributions

Dr. David G Sweet was responsible for drafting and revising the manuscript. Profs. Gerry HA Visser and Mikko Hallman prepared the first draft of the “Prenatal Care” section and assisted with subsequent revisions. Profs. Katrin Klebermass-Schrehof and Arjan te Pas performed literature searches and early drafts of the “Delivery Room Stabilisation” section as well as overall manuscript revisions. Profs. Christian P Speer, Charles Roehr, and Dr. David Sweet performed literature searches and early drafts of the “Surfactant Therapy” section as well as overall manuscript revisions. Profs. Ola Saugstad and Maximo Vento performed literature searches and provided early drafts of the “Oxygen beyond Stabilisation” section as well overall manuscript revisions. Profs. Gorm Greisen and Charles C Roehr provided early drafts of the “Non-Invasive Respiratory Support” section as well as overall manuscript revisions. Profs. Eren Ozek, Richard Plavka, and Arjan te Pas provided literature searches and early drafts of the “Mechanical Ventilation” section. Profs. Umberto Simeoni, Virgilio P Carnielli, and Eren Ozek provided literature searches and early drafts of the “Supportive Care” section. Dr. David Sweet and Prof Mikko Hallman drafted the miscellaneous considerations section. Professor Henry L. Halliday contributed to final editing and shortening of the manuscript.

## Figures and Tables

**Table 1:** Representations of quality of evidence and strength of recommendations

Quality of evidence
High quality	A
Moderate quality	B
Low quality	C
Very low quality	D
Strength of recommendation	
Strong recommendation for using intervention	1
Weak recommendation for using intervention	2

**Table 2:** Summary of recommendations

Prenatal care	Mothers at high risk of preterm birth < 28-30 weeks should be transferred to perinatal centres Offer a single course of prenatal corticosteroids to all women at risk of preterm delivery, from when pregnancy is considered viable up to 34 weeks of gestation. A single repeat course of steroids may be given in threatened preterm birth before 32 weeks of gestation if the first course was administered at least 1–2 weeks earlier MgSO_4_ should be administered to women in imminent labour before 32 weeks of gestation Clinicians should consider short-term use of tocolytic drugs in very preterm pregnancies to allow completion of a course of prenatal corticosteroids and/or in utero transfer to a perinatal centre

Delivery room stabilisation	Delay clamping the umbilical cord for at least 60s, especially in stable preterm infants Use a T-piece device rather than bag and mask Stabilise spontaneously breathing preterm infants with CPAP. If apnoeic, start mask ventilation/inflations, at initial CPAP pressure of 6–8 cm H_2_O and peak inspiratory pressures 20–25 cm H_2_O Use an oxygen blender. Start with FiO_2_ of 0.30 for babies <28 weeks, 0.21–0.30 for those 28–31 weeks, and 0.21 for 32 weeks of gestation and above. Adjust FIO_2_ guided by pulse oximetry, aim for SpO_2_ of 80% or more by 5 min of age Intubation should be reserved for babies not responding to positive pressure ventilation via face mask or nasal prongs Plastic bags or occlusive wrapping, radiant warmers, and humidified gas should be used during stabilisation for babies <32 weeks of gestation to reduce the risk of hypothermia

Respiratory support and surfactant	Use natural surfactant given by LISA technique where possible. Laryngeal mask surfactant can be used for more mature infants >1.0 kg. If intubated for stabilisation <30 weeks, give surfactant Treat worsening RDS with surfactant when FiO_2_ > 0.3 on CPAP pressure ≥6 cm H_2_O or if lung ultrasound suggests surfactant deficiency. Consider lower FiO_2_ thresholds for very immature infants. A second and third dose of surfactant can be given if there is ongoing evidence of RDS Start CPAP or (s)NIPPV as soon as possible in all babies at risk of RDS. HFNC can be used as an alternative, provided centres can provide CPAP or NIPPV for those falling HFNC If MV is required, use lung-protective modes such as VTV or high-frequency oscillation ventilation. Minimise the duration of MV. When weaning from MV, tolerate moderate hypercarbia but maintain pH above 7.22. Use iNO only where there is strong evidence of pulmonary hypertension such as pre- and post-ductal saturation difference Spontaneously breathing babies on MV should be extubated to CPAP, FIFNC, or NIPPV immediately following surfactant. BIPAP confers no advantage over CPAP; however, (s)NIPPV can reduce need for ventilation or need for re-ventilation following extubation Use caffeine routinely in infants <32 weeks of gestation to minimise need for MV Consider low-dose dexamethasone to facilitate extubation in infants ventilated > 1–2 weeks Oxygen saturation target should be between 90 and 94% with alarm limits 89% and 95%

Supportive care	Maintain body temperature between 36.5°C and 37.5°C at all times Start parenteral nutrition from birth, initial starting fluids around 80 mL/kg/day, restrict sodium intake during the first few days Start enteral feeding with mother's milk from day 1 if the baby is stable Use antibiotics judiciously and stop early when sepsis is ruled out Monitor blood pressure regularly, aim for normal tissue perfusion, use inotropes where deemed necessary (ECHO advised), and maintain haemoglobin within acceptable levels
